# Temporal patterns of weekly births and conceptions predicted by meteorology, seasonal variation, and lunar phases

**DOI:** 10.1007/s00508-022-02038-7

**Published:** 2022-05-24

**Authors:** Sandra Gudziunaite, Hanns Moshammer

**Affiliations:** 1 Department of Environmental Health, ZPH, Medical University of Vienna, Kinderspitalgasse 15, 1090 Vienna, Austria; 2 Department of Liberal Arts and Sciences, BASc, University College London, London, UK; 3 Department of Hygiene, Medical University of Karakalpakstan, Nukus, Uzbekistan

**Keywords:** Birth and conception rates, Time series study, Temperature, Annual and lunar cycles, Vienna

## Abstract

**Background:**

Data reporting the number of births given by women resident in Vienna for each week between 1999 and 2019, and the duration (in weeks) of their pregnancies, were used to estimate the week of conception. When preliminary examinations detected cyclical patterns for births and conceptions, a series of Poisson regressions for births and conceptions were performed to assess whether these cycles could be associated with lunar or solar phases.

**Methods:**

To that end 2 sine-cosine functions, 1 with a wavelength of 1 year (365.25 days) and 1 with a wavelength of 29.529 days, the latter resembling the average length of a lunar cycle, were constructed. In addition, also average weekly temperatures were included in the models.

**Results:**

Same week temperature was a strong non-linear predictor for both births and conceptions. Extreme temperatures, especially hot temperatures, increased the number of births while the numbers of successful conceptions were decreased by extreme temperatures. Regarding annual variation in conceptions, the lowest values were found in May and the highest in late autumn and early winter. Therefore, the highest numbers of births are observed in the summer. As per lunar variations, the highest numbers of conceptions occurred at the full moon and the highest numbers of births at the waxing crescent to first quarter moon.

**Discussion:**

The latter results should be treated with caution, as they are not hypothesis driven. Furthermore, literature reports on this topic are conflicting. Nevertheless, these findings will be useful in further analyses examining air pollution effects.

## Introduction

Folk wisdom wants us to believe that the moon phases affect birth numbers [1]. Scientific evidence regarding this claim is conflicting [2–4]. There seems to be no clear and strong biological mechanism linking lunar cycles to human pregnancy, nor can we imagine any strong evolutionary benefit in such a link. In epidemiological research we are rather interested in external influences that are caused by humans or can at least be mitigated by human action, such as air pollution. Our team has repeatedly investigated the effect of air pollution on risk of death in time series analyses [5, 6]. We felt the time was ripe to examine its impact on the other end of life.

Air pollution causes oxidative stress and systemic inflammation. We hypothesized that this would particularly affect early pregnancy shortly after conception [7]. Therefore, we needed data on births including information on the duration of pregnancy. From the Austrian Statistics Institute (Statistik Austria) we obtained anonymous data of all births from 1999 until 2019 from mothers with a home address in Vienna, assuming the home address of the mother would be the best determinant of the time course of early pregnancy exposure to air pollution. We restricted our data to births that had occurred in Austria, because births outside of Austria were only reported to Statistik Austria through cross-linkage with the citizens register starting in 2014, and these births usually lacked information about duration of pregnancy, as this information is not stored in the citizens register.

Time series analysis must control for time-varying confounding variables [8]. Therefore, we tried to understand the temporal structure of births before considering the impact of air pollution. This analysis was not hypothesis driven, but still we feel the results deserve separate reporting.

Extreme temperatures cause bodily stress in humans [9, 10]. Very low temperatures increase the systemic inflammatory state, while high temperatures increase the risk of dehydration and affect the electrolyte balance. So both stressors might increase the risk of preterm birth in pregnancies that already are vulnerable due to other causes. Therefore, extreme temperatures some weeks before term, would bring forward some births causing a reduction of births at term a few weeks later. We therefore would expect a negative impact of extreme temperatures on birth rate with a lag of several weeks; however, with zero lag we would expect a higher birth rate due to extreme temperatures, because extreme temperatures will not extend pregnancy time or prevent term births but will increase the number of preterm births instead.

Conception rates are most probably affected by human behavior in relation to sexual intercourse. It is perceivable that after a very hot or a very cold day, sexual desires might be weakened. Even though most sexual intercourse will occur indoors where people are less affected by outdoor temperatures, stressful temperatures over the course of the day will likely reduce nighttime activities. Therefore, we would expect fewer conceptions at extreme temperatures. In the literature though, evidence is stronger for the effect of high temperature [11, 12].

Annual variation in birth rates is well known [13–18]. Usually, the least numbers of successful intercourse occur in spring, while late autumn and early winter months are most productive, leading to highest birth rates in summer. In agricultural societies of old, and maybe also in Paleolithic hunter-gatherer tribes, nutritional status was undoubtedly best during summer, rendering that season the best choice for bringing up newborn children. Maybe, on the other hand, the dire and eventless long evenings and nights of the beginning of winter, provided little distraction from sexual activities; however, while numerous studies reported seasonal variations in birth rates, the seasonal pattern of births differs by geographical region [17, 19, 20], historical period [21], or sociodemographic factors [13, 18]. Not surprisingly, many different biological explanations are presented for the seasonality of birth rates, ranging from changes in food availability [22] to temperature effects [23] on sperm quality and to sunlight effects on hormonal regulation [14]. Most explanations focus on the time of conception examining both the male [24, 25] and the female role [26] in fecundability.

## Material and methods

### Data acquisition

Although data were obtained in anonymous format, there is always concern about too detailed information due to data privacy considerations. Also, information about pregnancy duration is only provided as pregnancy weeks. Therefore, we only obtained the week and not the exact day of birth from the Austrian birth register. We received data on both live and stillborn births with information on live status and about twin births and gender of the child included in the obtained data. In this first examination of the data this additional information was neglected though. Pregnancy duration is reported as number of weeks since the last menstrual bleeding. Since conception occurs roughly 2 weeks after the last bleeding, we calculated conception date by subtracting the pregnancy duration minus 2 from the week of birth.

We restricted our analysis to the 20 years 1999–2018 because from these years we were sure to have complete coverage both for birth and for conception dates, while in 2019 conception dates were necessarily incomplete.

Originally, we obtained daily average temperatures at the meteorological station “Hohe Warte” from the Zentralanstalt für Meteorologie und Geodynamik (ZAMG) and averaged these over the 7 days of every week, Next, we calculated the square of that weekly temperature minus 11.7 °C (the overall average temperature) and finally, we also added longer term averages (2 or 3 weeks) of temperature [27] and weekly averages of relative humidity to the model. “Hohe Warte” is situated in the hilly western outskirts of Vienna. Temperatures are there usually lower than in the city center, but we have demonstrated before [28] that daily temperature data at “Hohe Warte” and city center (“Stephansplatz”) are highly correlated with each other temporally.

### Preliminary analyses

Seasonality in the number of births and conceptions has first been explored preliminary using decompose and stl packages in R software [29] to perform seasonal and trend decomposition. LOESS smoother has been chosen amongst other default options offered because it does not automatically account for calendar holidays, can handle any type of seasonality, and is flexible to variations in seasonality rates.

### Statistical methods

When, apart from a long-term increasing trend, we saw clear evidence of two cycles, one the length of a year and the other of about 4 weeks, we decided to examine these cycles in more detail. We assumed that the shorter cycle was linked to the moon phases. Therefore, we constructed 2 sine-cosine functions, 1 with the wavelength of 1 year (365.25 days) and 1 with a wavelength of 29.529 days. The duration of a lunar month varies by 1 or even 2 days due to the fact that both earth and moon move on ellipses, not circles. But over the whole observation period the duration of 29.529 days turned out to be the best approximation of the average.

The sine-cosine functions were defined such that for the annual cycle the sine part of the function was closest to zero and the cosine part closest to 1 on each 31 December. For the lunar cycle the sine part was approximately −1 and the cosine part was zero at each full moon. At each new moon the sine part was approximately 1 and the cosine part was again zero. The cosine part reached its maximum (1) at the third quarter moon. The sine and cosine values of each Monday were used to represent the weekly values.

Initially, we examined negative binomial regression models. But, when we saw that overdispersion was negligible, we reverted to Poisson regression models. We ran separate but similarly constructed Poisson regression models for weekly number of births and number of conceptions. The first model each contained a linear term (weeks since 1 January 1999) and the two sine-cosine functions as independent variables. In a second model we also included temperature at the date of interest (either birth or conception). Since from air pollution studies on mortality we are familiar with a U-shaped non-linear association with temperature, we modelled temperature by a quadratic polynomial. In a sensitivity analysis, we assessed also each of the variables (linear time trend, lunar and solar cycles, and temperature) separately because we were concerned that the effect for example of temperature was a spurious one only due to smoothing out the misfit of the seasonal patterns. When we saw significant effects of temperature in the quadratic polynomial model, for visualization purposes we also performed a general additive model (GAM) using natural splines. We optimized the degrees of freedom for the temporal variation by minimizing the sum autocorrelation of the residuals [30]. The temperature effect was modelled as a natural spline with 3 degrees of freedom. The regression analyses were run in STATA (Vers. 16; STATACorp LLC, College Station, TX, USA) [31].

## Results

**Fig. 1:**
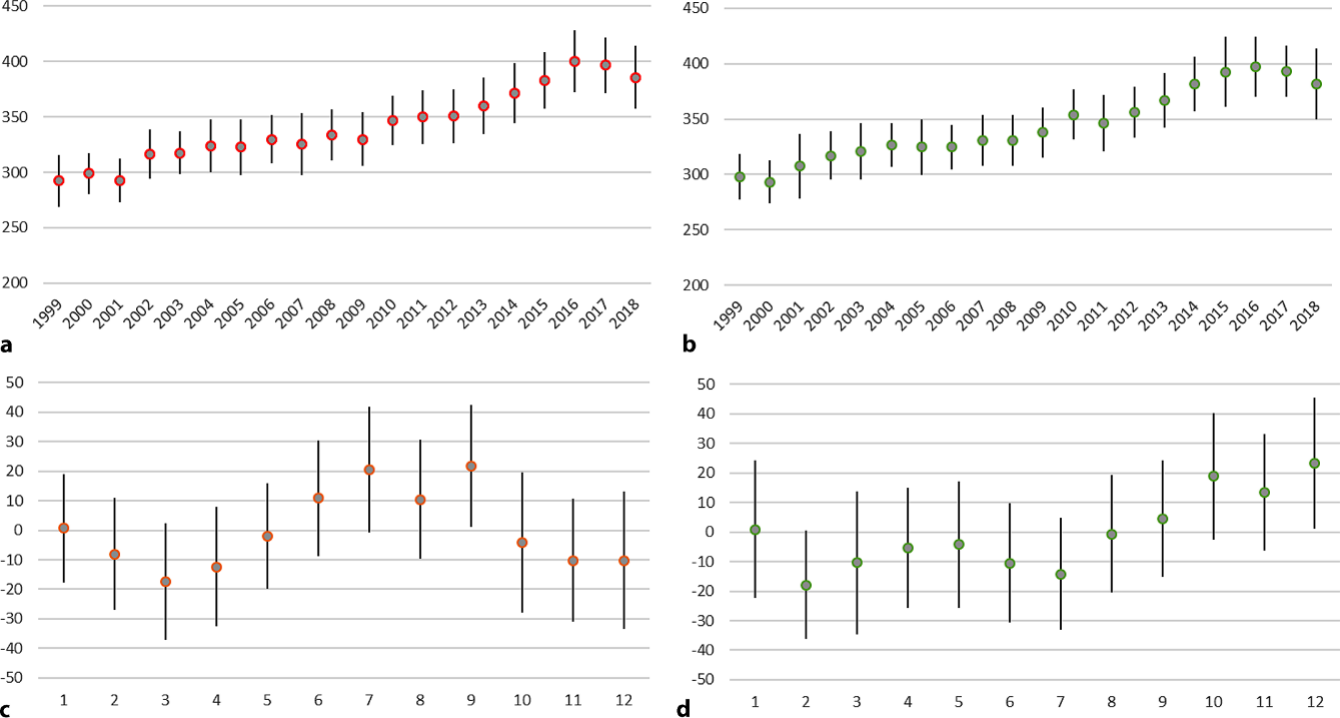
Number of weekly births (**a**) and conceptions (**b**) per year, mean ± standard deviation. For depicting the seasonal variation, the difference between weekly numbers and the annual average was calculated and that difference was presented per month for births (**c**) and conceptions (**d**)

In 1044 weeks there occurred on average 341.4 births (minimum: 235, maximum: 473) and 344.1 conceptions (221; 481). Average weekly temperature was 11.7 °C (−13.1; 29). The number of births and conceptions increased over the years and within each year some seasonal variation was visible (Fig. 1).

In all negative binomial regression models, alpha was less than 0.01 and therefore only the simpler Poisson regression models are reported.

Long-term trend, annual and lunar cycle, and same week temperature were all significantly associated with the numbers of birth and of conception (Tables 1 and 2). According to the first model number of births and of conceptions increased significantly over time (*p* < 0.001 each) and the linear trend accounted for most of the explained variation. Also, the sine and cosine parts of the annual cycle were highly significant. Birth numbers according to this smoothed model were lowest in January and February and highest in July and August. The number of conceptions were lowest in May and highest in November.

**Table 1:** Predictors of weekly number of births according to Poisson regressions

Variable	Univariate model			Multivariate model	
	Coefficient	Pseudo R^2^	*P*-value	Coefficient	95% Conf. Int
Week	0.0004	0.113	< 0.001	0.00030	0.00029; 0.00031
Sin (solar)	−0.029	0.014	< 0.001	−0.01826	−0.02350; −0.01301
Cos (solar)	−0.036		< 0.001	−0.02155	−0.03210; −0.01100
Sin (lunar)	0.002	0.002	0.459	0.00100	−0.00365; 0.00564
Cos (lunar)	−0.009		0.004	−0.00710	−0.01174; −0.00245
Temperature	0.004	0.037	< 0.001	0.00120	0.00028; 0.00212
Temp. squared	0.0001		< 0.001	0.00014	0.00009; 0.00018

**Table 2:** Predictors of weekly number of conceptions according to Poisson regressions

Variable	Univariate model			Multivariate model	
	Coefficient	Pseudo R^2^	*P*-value	Coefficient	95% Conf. Int
Week	0.0003	0.092	< 0.001	0.00030	0.00029; 0.00031
Sin (solar)	−0.0310	0.034	< 0.001	−0.02689	−0.03209; −0.02169
Cos (solar)	0.0316		< 0.001	0.01795	0.00745; 0.02844
Sin (lunar)	−0.0075	0.001	0.019	−0.00502	−0.00965; −0.00040
Cos (lunar)	−0.0027		0.408	−0.00016	−0.00479; 0.00447
Temperature	−0.0015	0.008	< 0.001	−0.00109	−0.00201; −0.00016
Temp. squared	−0.0001		< 0.001	−0.00011	−0.00015; −0.00006

Adding a sine-cosine function for the lunar cycles did not add much explanatory value and the coefficients were small. Nevertheless, the cosine part was significant for birth numbers (*p* = 0.003) and negative, while the sine part was approximately zero, indicating the highest birth numbers to occur roughly between the new and the full moon (waxing crescent to first quarter moon). According to the Akaike information criterion [32], but not the Bayesian information criterion [33], model performance improved slightly upon addition of the trigonometric function for the lunar cycle. For conception numbers, the sine part of the lunar cycle was significant (*p* = 0.038) and negative, while the cosine part was practically zero, indicating the highest number of conceptions occurring at the full moon.

**Fig. 2:**
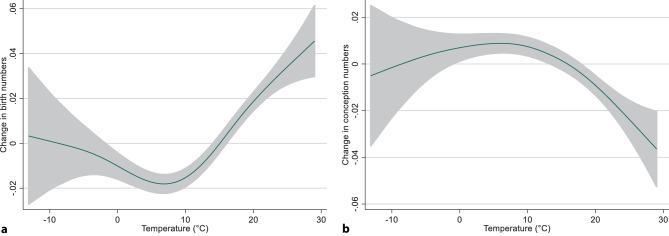
Natural spline with 3 degrees of freedom describing the temperature effects on **a** birth and **b** conception numbers

Temperature (both linear and even more so quadratic term) at the time of birth and conception, respectively, had also a highly significant effect on birth and conception numbers, but neither increased the explained variance much nor substantially changed the effect estimates for the two cycles. A positive coefficient for the quadratic term in the model explaining birth numbers indicated a U-shaped association between temperature and births. With conceptions a negative coefficient of the quadratic term was found indicating lower conception numbers at extreme temperatures. Both findings were confirmed in a GAM with a natural spline (Fig. 2). Chronic temperature (past 2 or 3 weeks) and relative humidity did not have any significant additional impact and were therefore not considered further.

## Discussion

Long-term, seasonal trends and meteorological conditions (and day of the week in the case of daily data) must be controlled for in time series analyses of air pollution, because these factors strongly affect mortality risk [8, 30] and likely also birth and conception numbers and are correlated with air quality.

Since day of the year and temperature do not display a linear association with mortality, some non-linear smoothing functions (e.g. spline models or LOESS) must be applied. It is vital to ensure that smoothing is optimized in order to neither cause residual confounding nor overadjustment. Several methods have been proposed to find the optimal smoother [8, 30]. In order to make a better informed choice of smoothing methods, we set out to study temporal and meteorological effects on the rates of birth and conception. Not unexpectedly, the unadjusted birth counts showed no strong signs of overdispersion (alpha in negative binomial regression being 0.01), as one would expect in a dataset that is mostly affected by random variation.

Controlling for temporal and meteorological factors reduced overdispersion even more with an alpha in negative binomial regression being around 0.001 in the full model. Generally, it is encouraging that these factors do not play an important role in birth and conception rates. This might render studies on air pollution effects less complicated. But still, to our surprise, some temporal and meteorological effects were found that warrant further discussion and closer examination.

### Temperature effects

As theoretically expected, a positive effect estimate for temperature squared on births was found, indicating that both hot and cold temperatures in late pregnancy could induce labor. For a deeper understanding of this phenomenon, temperature effects on pregnancy duration and not just birth counts should be studied as well. Especially the issue of low temperature effects deserves further research as several studies did not find an effect [34, 35], while Wu et al. [36] reported strongest effects of high diurnal variation in temperature on preterm birth rates. In comparison, effects of high temperatures on preterm delivery are much better established [37].

We expected fewer conceptions at extreme temperatures, which indeed was the case in our dataset. Although evidence from previous studies is stronger for the effect of high temperature [11, 12], temperature effects were significant both in the univariate and the complete model indicating that these findings reflect true and causal associations.

### Annual variation

Contrary to what we are told by numerous folk songs May is not the month of love. On the contrary, our data indicate that the least numbers of successful intercourse occur in that month, while late autumn and early winter months are most productive, leading to highest birth rates in summer. These findings are mostly in line with previous findings [13–18]. It is noteworthy that even in modern times with ample food supply during all times of the year as well as never-ending occupational and recreational opportunities distracting us from our sexual desires, these ancient patterns still hold, even though maybe to a lesser degree than historically.

### Lunar influences

We had not expected any variations with a cycle length approximating a lunar month. Most studies do not find lunar influences on birth rates [2–4, 38–42]. But some authors argue there is an association there [43–45], although sound biological explanations are often lacking and there is no clear consistency regarding the exact pattern in relation to the lunar phases. Nevertheless, there is some evidence for lunar influences on domestic animals [46] and wildlife [47, 48] also. So, even if a consistent biological mechanism is still lacking, the issue of lunar influences on life warrants further research.

We found the highest frequency of conceptions around the full moon. Because we estimated the conception data as 2 weeks after the last menstrual bleeding, that bleeding would then have occurred around the new moon. This finding is consistent with Law [44]. A recent study by Matsumoto and Shirahashi [45] is especially interesting as it does report no clear lunar pattern overall, but a significant lunar influence (with the largest number of births around the full moon) for those births that occurred at night, while births during daytime were more frequent around the new moon. Naturally, there seems to be a nocturnal pattern of births in humans, while births during daytime are predominantly triggered by medical interventions [49]. If nighttime births are “natural” and these natural births do occur more often near the full moon, then maybe the evolutionary sense in that lunar pattern was better visibility during moonlit nights. Still, the evolutionary sense in nighttime births is not quite clear. Females were certainly more vulnerable when giving birth. But it would be difficult to say if there were really fewer predators on the hunt during hunter-gatherer times at night. Again, the examination of our relatives in the animal kingdom might shed light on that issue [50]. While primates in wildlife give birth at night, when in captivity they switch to daytime delivery. If diurnal patterns and effects of lunar cycles are linked together (light at night), then changing diurnal patterns due to industrialization and hospitalization of births, and artificial light at night, will strongly affect lunar influences. If this is the case, our finding of higher birth rates at the waxing moon should not be over-interpreted, especially as we did not differentiate between daytime and nighttime births, or between natural or induced births.

Out of pure curiosity we also applied the sine-cosine cycle of 29.529 days to all-cause mortality data from Vienna 1970–2018 from our NO_2_ Study [6]. In the complete Poisson model lunar phases had no influence on daily mortality (*p*-values 0.58 for the sine part and 0.35 for the cosine part). If specificity is indeed a criterion for causality [51], this would support the assumption of an effect of lunar cycles on conception and birth.

### Strengths and limitations

This analysis was not driven by any a priori hypothesis. Therefore, *p*-values should be treated with caution. We examined birth and conception data over 20 years in a city with nearly 2 million inhabitants. That amount of data allowed for strong statistical power, enabling also the demonstration of small effects that need not have much biological relevance. We had no data on nighttime or daytime births nor on circumstances of birth like induced birth or cesarean section. And we only had weekly data. The latter might even have reduced statistical noise and the growing impact of the day of the week [1, 39] rendering smaller effects more visible. If, indeed, an effect of the lunar phases is real, this would most likely be acting through the brightness of the night sky. Now, looking at the (nearly) full moon at night, I would be hard pressed to say whether tonight is exactly full moon or if the full moon is to come in the next 1 or 2 days or did already take place, let alone judging it by the brightness alone. Therefore, we would expect an effect not of a single day (say: full moon), but rather of a couple of days. This might be better represented by weekly numbers. On the other hand, when weeks move through the lunar phases, some weeks will align nearly perfectly with the effective lunar periods, while at other times the match will not be so good. Therefore, we would still expect broader confidence intervals. This effect is, in our data set, partly set off by a relatively long observation period. The uncertainty is even larger for conception numbers, where the imprecision of the day of birth and of the gestational length is combined.

## Conclusion

While examining the temporal shape of weekly birth and conception data in preparation of a time series analysis on the effects of air pollution, we observed small quasi-monthly cycles. We tested the hypothesis applying a sine-cosine function with the average wavelength of the lunar cycle (29.529 days) and found that this function significantly predicted small-scale variations in birth and conception numbers. The variation explained is small and likely not biologically relevant. Nevertheless, given the controversy regarding lunar influences on human biology, we still find it noteworthy.
